# The Role of circRNA-SETD2/miR-519a/PTEN Axis in Fetal Birth Weight through Regulating Trophoblast Proliferation

**DOI:** 10.1155/2020/9809632

**Published:** 2020-06-12

**Authors:** Dan Wang, Quan Na, Guiyu Song, Ying Wang, Yang Wang

**Affiliations:** Department of Obstetrics and Gynecology, Shengjing Hospital of China Medical University, Shenyang, Liaoning 110004, China )

## Abstract

Abnormal birth weight is the one of the major causes of adulthood diseases such as obesity, metabolic syndrome, cardiovascular disease, type 2 diabetes, and hypertension. Accumulating evidence has suggested that the placental trophoblast is one of the most important reasons that influence birth weight. Our previous study showed that miR-519a are correlated with low fetal birth weight through regulating trophoblast proliferation. To further clarify the detailed mechanisms on how it is regulated, we screened the placental-specific circular RNAs (circRNAs) via microarray assay. The result identified that circ-SETD2 was highly expressed in the placenta of the patients with fetal macrosomia compared with healthy donors. Furthermore, bioinformatic analyses and the luciferase reporter assay revealed that miR-519a possessing the binding sites for both circ-SETD2 and phosphate and tensin homolog was deleted on chromosome 10 (PTEN). Interestingly, upregulation of circ-SETD2 enhanced the proliferation and invasion of the human trophoblast-like cell line HTR8/SVneo cell. A parallel study performed by Western blotting showed that overexpression of circ-SETD2 reduced miR-519a levels and increased PTEN levels in HTR8/SVneo cells. Importantly, the enhancement of HTR8/SVneo cell activity by circ-SETD2 overexpression was nullified when the cells were cotransfected by circ-SETD2 and miR-519a, suggesting the involvement of the circ-SETD2/miR-519a/PTEN axis in trophoblast activity. Taken together, we illustrate the role of circ-SETD2, as an upstream signaling of miR-519a/PTEN, in placenta development via regulating trophoblast proliferation and invasion. These findings improve our understanding of the mechanisms of progression of fetal macrosomia and will guide future development of therapeutic strategies against the disease by targeting the circ-SETD2/miR-519a/PTEN axis.

## 1. Introduction

Abnormal birth weight is one of the major causes of adulthood diseases such as obesity, metabolic syndrome, cardiovascular disease, type 2 diabetes, and hypertension [1]. Accumulating evidence has suggested that placenta, as an essential organ between the fetus and the mother, strictly controls the birth weight in the uterus [2]. In addition to nutritional factors, the proliferation and invasion of the placental trophoblast play a critical role in the development, maturation, and aging of the placenta during pregnancy [3, 4].

MicroRNAs (miRNAs) are short noncoding nucleotides or RNAs which can pair with 3′-untranslated regions (UTRs) of mRNAs. miRNAs are specifically expressed in various types of cells and tissues, and they function in gene silencing or posttranscriptional inhibition [5]. Aberrant expression of miRNAs in the placenta has been linked with the pathogenesis of pregnancy complications [6] [7]. Currently, our group identified that eight placenta-expressed miRNAs are expressed during the first trimester [8]. Of these eight placenta-expressed miRNAs, four miRNAs have been clarified to be involved in the regulation of complete hydatidiform moles [9]. Our recent study showed that several miRNAs including miR-517a, miR-518b, and miR-519a are correlated with low fetal birth weight through regulating trophoblast proliferation [10], suggesting the regulatory functions of placenta-expressed miRNAs in trophoblasts.

Circular RNAs (circRNAs) are a novel family of noncoding RNA, which are different from typical linear RNA and characterized by the presence of a covalent bond linking the 3′ and 5′ ends via backsplicing [11]. As a new member of competing endogenous RNAs, circRNAs have been shown to act as microRNA sponges and RNA-binding protein-sequestering agents, resulting in participation in the governing gene expression [12]. Hence, we hypothesized that placenta-specific circRNAs may function as an upstream regulator of miRNAs regulating the development of the placenta and fetal growth via controlling trophoblast proliferation.

## 2. Materials and Methods

### 2.1. Clinical Samples

The placental tissues used for this study were provided by the Shengjing Hospital of China Medical University (Shenyang, Liaoning, China) during the period from 2014 to 2015 with the informed consent of the patients, and all experiments were approved by the Ethics Committee of Shengjing Hospital of China Medical University (No. 2014PS86J). All placental tissues were collected from the donors of whom those with common pregnancy complications were excluded. The placental tissues were divided into two groups according to the newborns' birth weight: the macrosomia group (birth weight ≥ 4 kg, *n* = 25) and the control group (2.5 kg ≤ birth weight < 4 kg, *n* = 25). We chose a total of 8 samples including 4 patients with macrosomia and 4 controls for microarray sequencing. And we used a total of 50 samples including 25 patients with macrosomia and 25 controls for qRT-PCR confirmation. The detailed information of the patients and newborns is shown in Table 1.

Immediately after childbirth, the placenta tissues were collected from the maternal side of the placenta about 2 cm away from the umbilical cord insertion site, and both the infarction area and the calcification area were avoided. To remove the blood from tissues, the collected placental tissues were rinsed with sterilized saline for 5 times. These tissues were snap frozen in liquid nitrogen and stored at -80°C for analysis.

### 2.2. Microarray circRNA Processing and Data Analysis

The microarray assay was performed by using an Arraystar Human LncRNA V4.0 system (KangChen Bio-tech Inc., Shanghai, China). This array encompasses 20730 transcripts and variants and represents roughly 40173 lncRNAs. The cRNA preparation and labelling, hybridization, and scanning of microarrays were performed by One-Color Microarray-Based Gene Expression Analysis according to the manufacturer's protocol (Agilent Technology, Santa Clara, CA, USA). Briefly, the mRNA samples were first isolated via removing rRNA by a mRNA-ONLY™ Eukaryotic mRNA Isolating Kit (EPICENTRE Biotechnologies, Madison, Wisconsin, USA), followed by fluorescent labeled-cRNA synthesis and amplification through a T7 RNA Polymerase Blend reverse transcription system and Low Input Quick Amp Labeling Kit, respectively. After cRNA purification by RNeasy Mini Kit, the labeled cRNA was hybridized with a 2x Hi-RPM Hybridization Buffer while rotating at 10 rpm for 17 hours at 65°C. Subsequently, the microarray was washed with a Triton X-102 Wash Buffer and Gene Expression Wash Buffer and scanned by an Agilent DNA Microarray Scanner (G2505C, Agilent Technology). All reagents and buffers as mentioned above were provided by Agilent Technology.

The microarray data were extracted using Agilent Feature Extraction software 11.0.1.1(Agilent Technology), and the gene expression of placental tissues was calculated by GeneSpring GX 12.1 software (Agilent Technology). Fold change values for circRNAs were analyzed as the ratio of the signal values of the macrosomia group compared with the control group.

### 2.3. Cell Culture

Human trophoblast-like cell line HTR8/SVneo cells (ATCC, CRL-3271) were cultured in a Roswell Park Memorial Institute (RPMI) 1640 medium (Wako, Osaka, Japan) supplemented with 10% heat-inactivated fetal bovine serum (FBS) (MP Biomedicals, Santa Ana, USA) and 1% penicillin-streptomycin (Nacalai Tesque, Kyoto, Japan). For the luciferase reporter assay, human embryonic kidney cell line HEK293T cells (ATCC, CRL-11268) were cultured in Dulbecco's modified Eagle medium (DMEM, Wako, Osaka, Japan) with the same supplements as the RPMI 1640 medium. Both types of cells were grown in a 5% CO_2_ humidified incubator at 37°C.

### 2.4. Cell Transfection

HTR8/SVneo cells were cultured in antibiotic-free PRMI 1640 by changing the medium. After overnight incubation, the miR-mimics (50 nM) and miR-inhibitors (50 nM) as well as vectors (50 nM) were transfected into HTR8/SVneo cells using Lipofectamine 3000 (Invitrogen, Waltham, MA, USA) according to the manufacturer's instructions. To confirm the miR-519a as a downstream target of SETD2, SETD2 was cotransfected into HTR8/SVneo cells with miR-519a mimics. The miR-mimic or miR-inhibitor for miR-519a and the mimic negative control (miR-NC) were designed by Biomics Biotech (Jiangsu, China). Human SETD2 vector was purchased from Applied Biological Materials Inc. (PV430741, Richmond, BC, Canada). An empty vector, as the negative control, was also provided by Applied Biological Materials Inc.

### 2.5. Quantitative Real-Time PCR (qRT-PCR)

Total RNAs from tissues or cells were isolated by a TRIzol Reagent (Invitrogen, Waltham, MA, USA), followed by the reverse transcription of extracted RNAs through a miScript II RT kit (QIAGEN, Hilden, Germany) in a fluorescence thermal cycler (Bio-Rad Laboratories, Inc., Hercules, CA, USA). The expression of relative genes was measured by a SYBR Green PCR reagent kit (Applied Biosystems) with the following primer sets on an ABI ViiA7 Real-Time PCR System. The relative expression of target genes was normalized to *β*-actin. The biological information and primer sequences are shown in Table 2 and supplementary Figure 1, respectively.

### 2.6. Luciferase Reporter Assay

The fragments of the 3′UTR of SETD2 and PTEN containing miR-519a binding sites and its mutants were amplified by PCR, followed by the insertion of products into pmirGLO dual-luciferase miRNA target expression vector (Promega, Madison, WI, USA). HEK293T cells were transfected with those reporter vectors and a control vector by Lipofectamine 3000 (Invitrogen, Waltham, MA, USA). After transfection for 48 h, the relative luciferase activity was detected by the Dual-Luciferase Reporter Assay Kit (Promega, Madison, WI, USA) and normalized by Renilla luciferase according to the manufacturer's protocol.

### 2.7. Transwell Invasion Assay

Matrigel (BD Biosciences, CA, USA) was diluted by FBS-free DMEM (cold) at the ratio of 1 : 8, followed by addition of the mixture into the polycarbonate membrane inserts of the transwell plate. Four-hour incubation was needed to turn the Matrigel into a gel. The transfected cells were cultured in FBS-free DMEM overnight. Next day, the cells were suspended in FBS-free DMEM at a density of 5 × 10^5^ cells/mL and plated in the polycarbonate membrane inserts. The 20% FBS-containing DMEM was added into the lower area of the transwell plate. To test the ability of cell invasion, the top chamber was removed after 24 h and crystal violet staining was performed.

### 2.8. Cell Proliferation

The proliferation ability of HTR8/SVneo cells was determined by Cell Counting Kit-8 (CCK-8 Kit, Dojindo, Kumamoto, Japan) based on the reduction of WST-8 to WST-8 formazan. In brief, the HTR8/SVneo cells were seeded in a 96-well plate at a density of 5 × 10^4^ cells/well. On the day of the experiment, the cells were transfected with an empty vector and circ-SETD2. Ten microliters of the CCK-8 reagent was added into the culture medium at the indicated time and incubated for 60 min, followed by the measurement of absorbance at 450 nm using a microplate reader (Bio-Rad, Hercules, CA, USA).

### 2.9. Flow Cytometry (FACS) Analysis

Annexin V-Alexa Fluor-488/propidium iodide (PI) staining was used to stain apoptotic HTR8/SVneo cells. After circ-SETD2 transfection for 48 h, HTR8/SVneo cells were firstly stained by Annexin V-Alexa Fluor-488 for 15 min on ice. The secondary staining was performed by adding PI solution. All experimental procedures were strictly protected from light. The data were calculated by FlowJo software (Tree Star, Ashland, OR) after FACSCalibur (BD Biosciences, CA, USA) analysis.

### 2.10. Western Blotting

The HTR8/SVneo cells were homogenized in a radioimmunoprecipitation assay buffer (RIPA, 25 mM Tris-HCl pH 7.6, 150 mM NaCl, 1% NP-40, 1% sodium deoxycholate, 0.1% SDS). A BCA Protein Assay Kit (FUJIFILM Wako Pure Chemical Corporation, Osaka, Japan) was used for determination of samples' concentrations. Fifteen micrograms of each sample was separated by SDS-PAGE. The proteins were transferred to polyvinylidene difluoride (PVDF) membrane (Immobilon-P; EMD Millipore, Darmstadt, Germany) proteins, and the membrane was blocked with 5% of skim milk for 1 h at room temperature. Targeted proteins were incubated with primary antibodies against SETD2 (PA5-34934, Thermo Scientific, Waltham, MA, USA) and PTEN (Cell Signaling #9559) overnight at 4°C. All primary antibodies were used at 1000 times dilution, and the GAPDH (Cell Signaling, #5174) was used for control. The secondary anti-rabbit antibody (Cell Signaling, #5127) was incubated at room temperature for 1 h with 5000 times dilution. Protein band intensity was analyzed by using a Luminata Forte Western HRP substrate (Millipore) with a Bio-Rad ChemiDoc XRS+ imaging system (Bio-Rad Laboratories, Hercules, CA, USA).

### 2.11. Statistical Analysis

In this study, the *χ*^2^ test was used to analyze the relation between the circ-SETD2 and miR-519a levels, the PTEN and miR-519a levels, and the circ-SETD2 and PTEN levels in macrosomia placental tissues and their clinicopathological characteristics. All experiments were conducted with at least three independent reproductions. The data are represented as the mean ± standard deviation (S.D.) using GraphPad Prism 5.0 (GraphPad Software, Inc., San Diego, Ca, USA). Statistical analyses were performed by using Student's *t*-test. Statistical significance was defined as a *p* value of less than 0.05.

## 3. Results

### 3.1. Identification of circ-SETD2 as a Novel Fetal Birth Weight-Associated Gene

**Figure 1:**
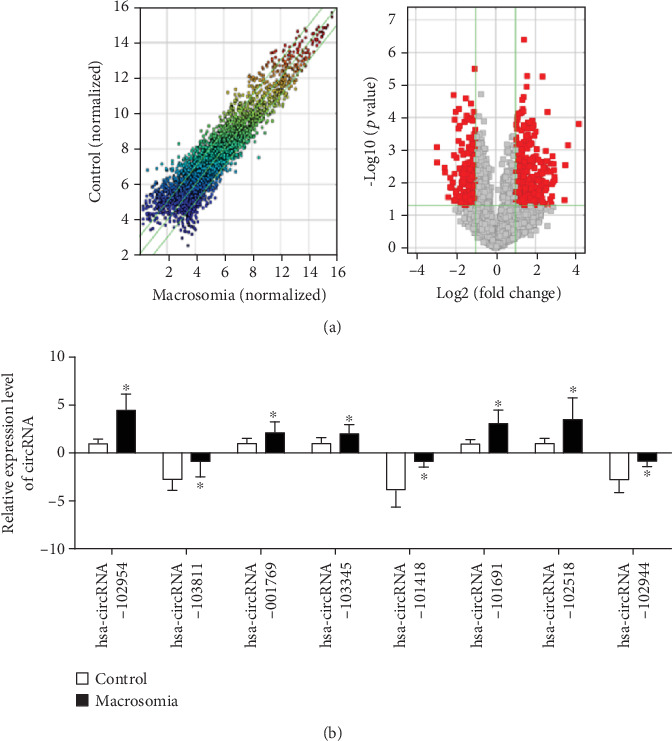
Identification of circ-SETD2. Volcano plot of circRNA expression (a). *x*-axis: log2 ratio of circRNA expression levels in placenta of normal and fetal macrosomia patients. *y*-axis: the FDR value (-log10 transformed) of circRNAs. The distribution of identified circRNAs in each chromosome (b). Relative gene expression was normalized by GAPDH expression. The data are represented as the mean ± S.D. (*n* = 3). ^∗^*p* < 0.05; ^∗∗^*p* < 0.01.

By analyzing the most differentially expressed circRNAs between fetal macrosomia donors and healthy donors (Figure 1(a)), we firstly identified a total of 20 circRNAs including 10 upregulated circRNAs and 10 downregulated circRNAs, in which expression levels in placentas of fetal macrosomia donors were at minimum 2 times different than those in placentas of healthy donors (Table 2). Base on this, we further selected 8 circRNAs, including 5 upregulated circRNAs and 3 downregulated circRNAs, and confirmed their expression in the placentas (Figure 1(b)). We then studied these circRNAs through TargetScan (http://www.targetscan.org/vert_72/) as well as miRanda (http://www.microrna.org/microrna/home.do), and combined with our previous studies [13], circ-SETD2 (hsa-circRNA-103345) was decided and used for a further experiment.

### 3.2. circ-SETD2 Regulates HTR8/SVneo Cell Proliferation and Invasion

**Figure 2:**
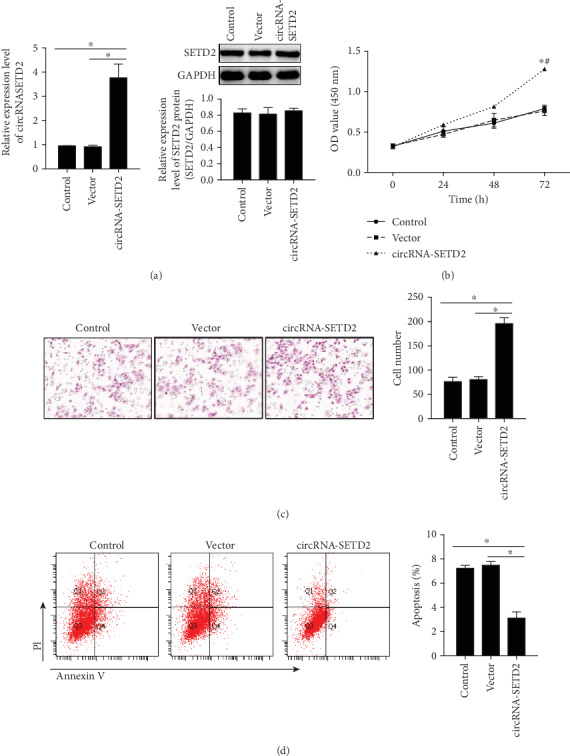
The effect of circ-SETD2 on trophoblast-like HTR8/SVneo cells. The HTR8/SVneo cells were transfected with circ-SETD2 vector. The mRNA levels and protein levels of circ-SETD2 in HTR8/SVneo cells were measured by qRT-PCR (a) and Western blotting (b), respectively. The proliferation of HTR8/SVneo cells was examined by CCK-8 kit post transfection (c). Transwell assay was performed to analyze the invasive activity of circ-SETD2-overexpressed HTR8/SVneo cells (d). The apoptotic HTR8/SVneo cells were analyzed using Annexin V/PI staining via FACS (e). Relative gene expression was normalized by GAPDH expression. The data are represented as the mean ± S.D. (*n* = 3). ns: not significant. ^∗^*p* < 0.05; ^∗∗^*p* < 0.01.

With the circ-SETD2 expression in the placenta of both fetal macrosomia patients and healthy donors now clearly quantified, we sought to clarify the effects of circ-SETD2 on placental development by using an *in vitro* trophoblast-like HTR8/SVneo cell culture system. As shown in Figure 2(a), transfection of HTR8/SVneo cells with circ-SETD2 significantly increased in mRNA levels of circ-SETD2, but not in protein levels, indicating the circ-SETD2 but not messenger RNA. Importantly, upregulation of circ-SETD2 in HTR8/SVneo cells enhanced cell proliferation (Figure 2(b)) and invasive activity (Figure 2(c)). It was also observed that the apoptotic HTR8/SVneo cell numbers were significantly decreased by circ-SETD2 overexpression (Figure 2(d)). These data provide evidence that the proliferation and invasion of trophoblast cells were regulated by circ-SETD2.

### 3.3. miR-519a/PTEN Is the Downstream Target for circ-SETD2

**Figure 3:**
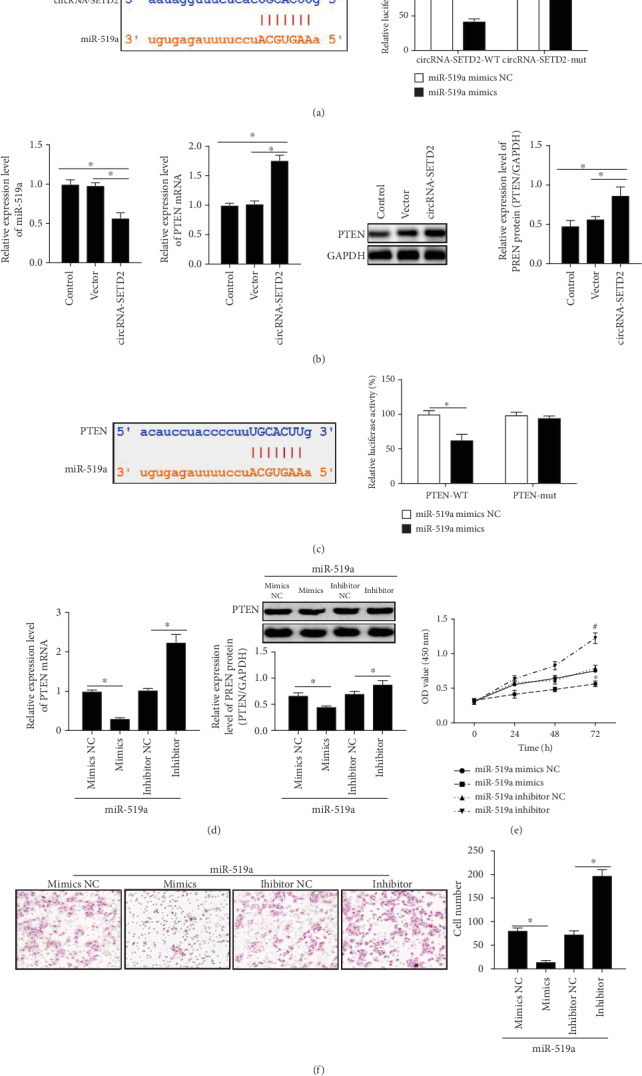
The enhanced cellular proliferation and invasion by circ-SETD2 are related to miR-519a/PTEN. miR-519a provides binding sites with circ-SETD2 and PTEN (a). The mRNA levels of miR-519a and PTEN were quantified by qRT-PCR, and Western blotting was used for analysis of protein expression of PTEN (b). The binding between miR-519a and PTEN was confirmed by luciferase assay (c). The mRNA levels and protein levels of PTEN in miR-519a-overexpressed or miR-519a-downregulated HTR8/SVneo cells were measured by qRT-PCR and Western blotting, respectively (d). The proliferation and invasive activity of HTR8/SVneo cells were respectively examined by CCK-8 kit (e) and transwell assay (f) after transfection of cells with miR-519a mimics or inhibitors. Relative genes expression was normalized by GAPDH expression. The data are represented as the mean ± S.D. (*n* = 3). ^∗^*p* < 0.05; ^∗∗^*p* < 0.01.

Since either TargetScan or miRanda predicted that miR-519a possessed the binding sites of both circ-SETD2 and PTEN (Figures 3(a) and 3(c)), we sought to understand the regulatory mechanism of circ-SETD2 on placental development by targeting on these molecules. As expected, upregulation of circ-SETD2 in HTR8/SVneo cells declined miR-519a levels and raised both PTEN mRNA levels and protein levels (Figure 3(b)). Moreover, the luciferase assay strongly proved that miR-519a binds to the 3′UTR of PTEN (Figure 3(c)). To further confirm the interaction between miR-519a and PTEN, the HTR8/SVneo cells were then transfected with miR-519a mimics as well as a miR-519a inhibitor (supplementary Figure 2). It was observed that transfection of HTR8/SVneo cells with miR-519a mimics inhibited PTEN transcription and translation. However, enhancement of PTEN mRNA levels and protein expression was observed when the HTR8/SVneo cells were transfected by the miR-519a inhibitor (Figure 3(d)). Simultaneously, it was observed that miR-519a mimic treatment caused lower cell proliferation and weaker invasive activity compared with control HTR8/SVneo cells. On the contrary, higher cell proliferation and stronger invasive activity of HTR8/SVneo cells were induced by miR-519a inhibitor transfection, as compared with control HTR8/SVneo cells (Figures 3(e) and 3(f)). These results indicated that miR-519a/PTEN function as the downstream target of circ-SETD2 during trophoblast cell proliferation and invasion.

### 3.4. circ-SETD2 Regulates HTR8/SVneo Cell Proliferation and Invasion through Targeting miR-519a/PTEN

**Figure 4:**
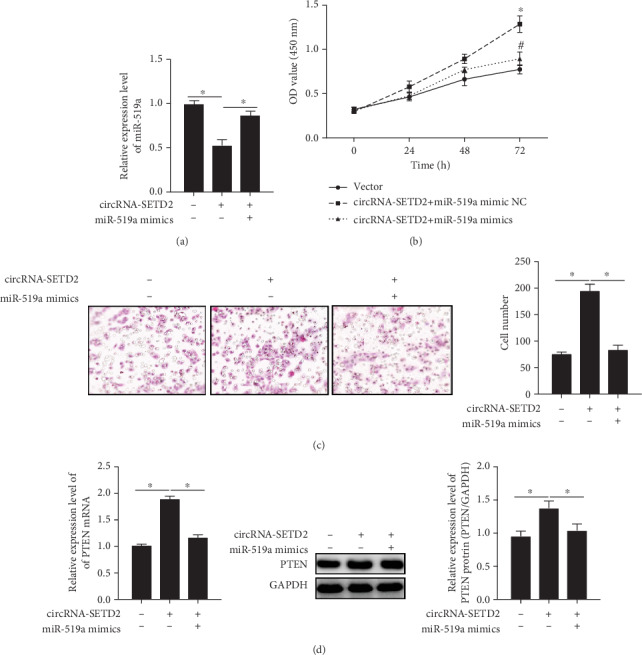
HTR8/SVneo cell proliferation and invasive activity were regulated by circ-SETD2/miR-519a/PTEN axis. The mRNA levels of miR-519a in cotransfected HTR8/SVneo cells were quantified by qRT-PCR (a). The proliferation of HTR8/SVneo cells after SETD2/miR-519a cotransfection was measured by CCK-8 kit (b). Transwell assay was used to examine invasive activity of SETD2/miR-519a-overexpressed HTR8/SVneo cells (c). The expression of PTEN was analyzed by Western blotting (d). Relative gene expression was normalized by GAPDH expression. The data are represented as the mean ± S.D. (*n* = 3). ^∗^*p* < 0.05; ^∗∗^*p* < 0.01.

To confirm the biological function of the circ-SEDT2/miR-519a/PTEN axis in trophoblast development, we established the circ-SETD2/miR-519a coexpressing HTR8/SVneo cells. By using this system, it was found that cotransfection of HTR8/SVneo cells with circ-SETD2 and miR-519a nullified circ-SETD2-induced downregulation of miR-519a (Figure 4(a)). Moreover, either proliferation or invasive activity of HTR8/SVneo cells was reduced when the cells were cotransfected by circ-SETD2 and miR-519a, as compared with circ-SETD2-overexpressing HTR8/SVneo cells (Figures 4(b) and 4(c)). Of note, the upregulation of PTEN by circ-SETD2 overexpression was also abrogated when the circ-SETD2 and miR-519a coexpressed in HTR8/SVneo cells (Figure 4(d)). It was illustrated that the circ-SETD2/miR-519a/PTEN axis regulates the fetal birth weight through control of the trophoblast proliferation.

### 3.5. circ-SETD2, miR-519a, and PTEN Are Correlated with Fetal Macrosomia

**Figure 5:**
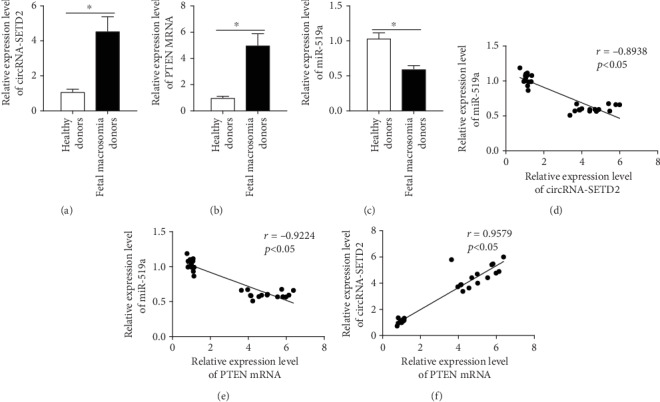
The mRNA levels of circ-SETD2, miR-519a, and PTEN in placenta of fetal macrosomia patients. The mRNA levels including circ-SETD2, miR-519a, and PTEN in placenta for fetal macrosomia donors and healthy donors were quantified using qRT-PCR (a–c), and the correlation between levels of those genes was analyzed using SPSS software (d–f). The data are represented as the mean ± S.D. (*n* = 25).

Finally, we examined the levels of circ-SETD2, miR-519a, and PTEN in placentas of fetal macrosomia patients and healthy donors. The aberrant high levels of circ-SETD2 and PTEN were found in the fetal macrosomia patients' group. Meanwhile, underexpression of miR-519a was observed in the fetal macrosomia patients' group (Figures 5(a)–5(c)). Spearman's correlation analysis showed significant inverse correlations of the miR-519a and circ-SETD2 and miR-519a and PTEN expression levels in placentas of fetal macrosomia patients (Figures 5(d) and 5(e)). In addition, a positive correlation of the PTEN and circ-SETD2 expression levels in the same tissues was clarified (Figure 5(f)). Taken together, these data again supported that the fetal birth weight is regulated by the circ-SETD2/miR-519a/PTEN axis.

## 4. Discussion

The pathogenesis of fetal macrosomia is a complex process, in which genetic, metabolic, and endocrine factors are involved [14]. However, the underlying mechanism requires further verification. In the present study, we provided evidence that the involvement of circ-SETD2 (circRNA_103345) in placenta increased in women with macrosomia. In terms of mechanism, circ-SETD2 can bind specifically to miR-519a, which controls the proliferation as well as invasion of trophoblasts via regulating the PTEN signaling. To our best knowledge, this is the first data showing the regulation of fetal birth weight by placenta-specific circ-SETD2.

As a medium connecting the mother and the fetus, the placenta is a critical organ to mediate fetal development. On the other hand, the trophoblasts are specialized cells of the placenta that possess a high proliferation activity during the first trimester of pregnancy, which becomes a key factor of placenta development [15]. Abnormal proliferation of trophoblasts has been linked with various pregnancy complications such as preeclampsia, intrauterine growth restriction, and unexplained miscarriage [16]. In this report, our data clearly showed that overexpression of circ-SETD2 by transfection increases the proliferation and invasion of HTR8/SVneo cells in a human trophoblast-like cell line (Figures 2(b) and 2(c)) and decreases the apoptotic cell number of HTR8/SVneo cells (Figure 2(d)), indicating that circ-SETD2 regulates the placental development via controlling biological behaviors of trophoblasts like proliferation and invasion as well as apoptosis.

The noncoding RNAs such as circRNAs and miRNAs have been discovered over 20 years ago [17]; their biological functions in the placenta are still poorly understood. Our previous data showed that miR-519a contributes to the pathogenesis of complete hydatidiform moles and fetal birth weight [9] [13], suggesting the involvement of miR-519a in trophoblast proliferation. Expectedly, the proliferation and invasion of HTR8/SVneo cells were strongly suppressed when the cells were transfected by miR-519a mimics and enhanced by treatment cells with the miR-519a inhibitor (Figures 3(e) and 3(f)). Furthermore, TargetScan and the luciferase reporter assay proved that miR-519a possesses the binding sites of both circ-SETD2 and PTEN (Figures 3(a)–3(d)). On this basis, the circ-SETD2/miR-519a/PTEN axis is considered to be a novel signaling of trophoblast proliferation and invasion. In agreement with our hypothesis, our data showed that enhancement of HTR8/SVneo cell proliferation and invasion by miR-519a inhibitor was nullified by cotransfection of the cells with circ-SETD2 (Figures 4(a)–4(c)), which is the powerful evidence that miR-519a is a downstream target of circ-SETD2.

A number studies indicate that PTEN is closely associated with the biological activity of trophoblasts such as proliferation, migration, and invasion [18] [19] [20]. Here, we found that PTEN positively regulates the trophoblast growth (Figures 3 and 4). More important, the PTEN level in HTR8/SVneo cells is increased by circ-SETD2 (Figure 3(b)). In contrast to circ-SETD2, miR-519a negatively regulates the PTEN expression in HTR8/SVneo cells (Figure 3(d)). In addition, it was also observed that enhancement of PTEN levels by circ-SETD2 is inhibited via cotransfection of HTR8/SVneo cells with circ-SETD2 and miR-519a (Figure 4(d)). Our findings identify that the PTEN, as a downstream signal of the circ-SETD2/miR-519a axis, regulates trophoblast activity.

Clinically, several criteria were proposed for the prediction of fetal macrosomia. For instance, sonographic prediction of weight at delivery bigger than 4000 g (8 lb, 13 oz) and 4500 g (9 lb, 15 oz) regardless of the gestational age or weight at delivery exceeding the 90^th^ percentile or above 2 standard deviations, for gestational age and ethnicity [21]. Besides, blood lipids, lipoproteins [22], glucose homeostasis [23], etc. have also been considered suitable indexes of fetal macrosomia in mothers who are not diabetic. For the pregnancies affected by diabetes, some glycaemic markers and hormones are used for macrosomia prediction [24]. However, the accurate prediction of fetal macrosomia remains a challenging goal. As per the data present in Figure 5, the dramatic enhancement of circ-SETD2 and PTEN and the reduction of miR-519a levels in patients with fetal macrosomia were observed. This finding highlights the impact of the circ-SETD2/miR-519a/PTEN axis as a predictive potential target for fetal macrosomia pathogenesis, although the accuracy and the prospect of predicting fetal macrosomia by targeting these molecules are currently not known and warrant further investigation.

Taken together, we illustrate the role of circ-SETD2 in the placental development via regulating trophoblast proliferation and invasion. Molecularly, the function of circ-SETD2 emerged via a restriction of miR-519a, resulting in the increase of PTEN. These findings improve our understanding of the mechanisms of pathogenesis of fetal macrosomia and will guide future development of therapeutic strategies against fetal macrosomia by targeting the circ-SETD2/miR-519a/PTEN axis.

## Figures and Tables

**Table 1:** The clinical information of fetal macrosomia patients and healthy volunteers.

	Macrosomia group (*n* = 4)	Control (*n* = 4)
Age (years)	33.25 ± 2.14	32.0 ± 2.61
BMI (kg/m^2^)	22.58 ± 1.56	21.21 ± 1.51
Gravidity	2.75 ± 0.85	3.0 ± 0.71
Parity	0.50 ± 0.29	0.75 ± 0.48
Gestational week	38.82 ± 0.95	38.86 ± 1.08
Smoking	0	0
Alcoholism	0	0
Newborn birth weight (g)	4290 ± 138.1^∗^	3135 ± 37.53

^∗^ *P* < 0.05

**Table 2:** The biological information of 20 identified circRNAs.

circRNAs	Chrom	Gene symbol	Regulation	FC (abs)	*p* value
hsa_circRNA_103052	chr20	C20orf24	Up	18.076257	0.0001555
hsa_circRNA_102518	chr19	UBA2	Up	12.329134	0.0007075
hsa_circRNA_101691	chr16	CREBBP	Up	11.265317	0.002884
hsa_circRNA_100904	chr11	ALG8	Up	10.952723	0.0342738
hsa_circRNA_102259	chr17	TBCD	Up	7.779316	0.0084235
hsa_circRNA_104566	chr8	PSD3	Up	7.7463712	0.0067648
hsa_circRNA_104591	chr8	ASH2L	Up	7.6168811	0.0041182
hsa_circRNA_104052	chr6	CDYL	Up	7.5173534	0.0026272
hsa_circRNA_104666	chr8	PABPC1	Up	7.366699	0.0093712
hsa_circRNA_100165	chr1	ZMYM4	Up	7.2240414	0.0015469
hsa_circRNA_101379	chr14	SMOC1	Down	7.7023711	0.0008131
hsa_circRNA_101418	chr14	CEP128	Down	7.6476965	0.0024176
hsa_circRNA_103856	chr5	ADAMTS6	Down	5.8961449	0.0034304
hsa_circRNA_102944	chr2	DIS3L2	Down	5.8333338	0.0048137
hsa_circRNA_104438	chr7	AP4M1	Down	5.2117737	0.0278173
hsa_circRNA_104298	chr7	EIF2AK1	Down	4.8526554	0.0138268
hsa_circRNA_103664	chr4	SDAD1	Down	4.8322185	0.0071268
hsa_circRNA_103569	chr3	LRCH3	Down	4.673573	0.0143129
hsa_circRNA_102566	chr19	RELB	Down	4.466375	0.0097179
hsa_circRNA_103225	chr22	DDX17	Down	4.3173615	2.036*E*-05

## Data Availability

The data used to support the findings of this study are available from the corresponding author upon request.

## Additional Points

*Highlights*. (1) circ-SETD2 is highly expressed in the patients with fetal macrosomia. (2) Upregulation of circ-SETD2 reduces the miR-519a levels and increases the PTEN levels in HTR8/SVneo cells. (3) circ-SETD2 overexpression enhances the proliferation and invasion of HTR8/SVneo cells and inhibits the HTR8/SVneo cell apoptosis. (4) The circ-SETD2/miR-519a/PTEN axis regulates the placental development via controlling trophoblast activity. (5) Fetal macrosomia is positively correlated with circ-SETD2 and PTEN and negatively correlated with miR-519a.

## Supplementary Materials

Supplementary materials Supplementary Figure 1: the primer sequences of 20 identified circRNAs. Supplementary Figure 2: the expression of miR-519a. The HTR8/SVneo cells were transfected with miR-519a mimics as well as a miR-519a inhibitor, followed by measurement of expression of miR-519a via qRT-PCR. The data are represented as the mean ± S.D. (*n* = 3). ^∗∗^*p* < 0.01.
