# Supplementary material for: The Role of circRNA-SETD2/miR-519a/PTEN Axis in Fetal Birth Weight through Regulating Trophoblast Proliferation
Source: Biomed Res Int. 2020 Jun 12;2020:9809632. doi: 10.1155/2020/9809632 (PMC7306081; doi:10.1155/2020/9809632)

Supplementary Figure 1

Gene name	Bi-directional primer sequence
**GAPDH**	F:5'GGGAAACTGTGGCGTGAT3'<br>R:5'GAGTGGGTGTCGCTGTTGA3'
**hsa_circRNA_102954**	F:5' GGGATTAAACTCTTTGTTTACTCC 3'<br>R:5' TATTTCCCCTGTCTCTGTGATG 3'
**hsa_circRNA_103811**	F:5' GAGCGAATAGAGAGAGAATCAGC 3'<br>R:5' CTGTCATTACTTCCCATTTCCC 3'
**hsa_circRNA_001769**	F:5' GTTAATGGAAGTGAACCAAGCA 3'<br>R:5' CAGGGAAATTCTGTAGGGTAAAG 3'
**hsa_circRNA_103345**	F:5' TGTTCCAATAGACGGTTTCAGAG 3'<br>R:5' GCACATTTTCAATCTTTGCCTC 3'
**hsa_circRNA_101418**	F:5' AAAGCACATTTGGCATCACA 3'<br>R:5'CTTCTCAGTTTCAGTTTGTCTTAGC 3'
**hsa_circRNA_101691**	F:5' GTGCTGGCTGAGACCCTAAC 3'<br>R:5' CAAGTCAAACAATGATCCAAAATC 3'
**hsa_circRNA_102518**	F:5' TACATTGCATCGTTTGGGC 3'<br>R:5' AACTGTCTGTTGAGGTTGCTTAC 3'
**hsa_circRNA_102944**	F:5' GAGGAGCATTGGAAGCGAA 3'<br>R:5' CTCAGAGTTCCCGCCTTGA 3'
**hsa_circRNA_103052**	F:5' ATTCTGCCTGATCAATGC 3'<br>R:5' AAACCCTTCCTTCGTGAG 3'
**hsa_circRNA_100904**	F:5' TTCCCGACTCTTTCCTTTC 3'<br>R:5' CGATGACAGACAGCACTT 3'
**hsa_circRNA_102259**	F:5' GTCGGTAACAGATCCAACT 3'<br>R:5' ACAAGGCGTAAGCAACTT 3'
**hsa_circRNA_104566**	F:5' GCCACGGACTATGAGAAG 3'<br>R:5' ACTTCAGTTGCTCCTCCT 3'

**hsa_circRNA_104591**	F:5’ ATGACAACCAGACAGAGAC 3’
	R:5’ AGCAGGACCAATGTTACTAA 3’
**hsa_circRNA_101379**	F:5’ AGTCTTTCTTCCCTTTCCTT 3’
	R:5’ CCGTGTAACATAAGTGGTG 3’
**hsa_circRNA_103856**	F:5’ AGGATTCAAGCATCGGATT 3’
	R:5’ CACACTGAGCAGAACACT 3’
**hsa_circRNA_104438**	F:5’ TTCATTCACATCAGACACAG 3’
	R:5’ ATACCAGAGCCACATTGC 3’
**hsa_circRNA_104298**	F:5’ GAAGTAGCCTTGGAAGCA 3’
	R:5’ TCCTGACCTTGTATACTCTTC 3’
**hsa_circRNA_103664**	F:5’ TATGGCACAGATTAGTCACT 3’
	R:5’ CAGGCTTGATGGATTGATG 3’
**hsa_circRNA_103569**	F:5’ CGTTATATTCCAGAGGCAAT 3’
	R:5’ CAAATGTCTAAGGTGTCCAA 3’
**hsa_circRNA_102566**	F:5’ GTGTGAGGAAGAAGGAGATT 3’
	R:5’ GTCGCAGAGCAAGTAGAG 3’

Supplementary Figure 2

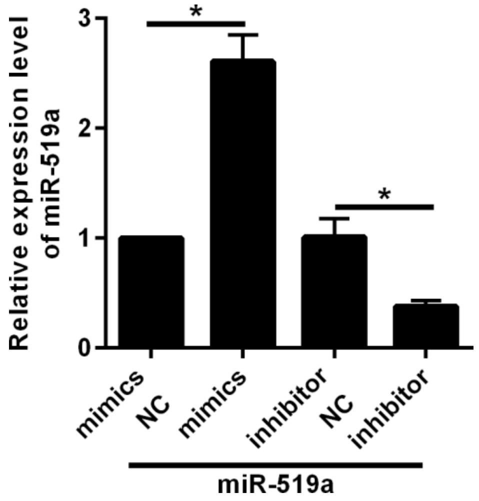

Supplement: Supplementary materials — Supplementary Figure 1: the primer sequences of 20 identified circRNAs. Supplementary Figure 2: the expression of miR-519a. The HTR8/SVneo cells were transfected with miR-519a mimics as well as a miR-519a inhibitor, followed by measurement of expression of miR-519a via qRT-PCR. The data are represented as the mean ± S.D. (n = 3). ∗∗p < 0.01. [file 9809632.f1.pdf]
